# Atlatl use equalizes female and male projectile weapon velocity

**DOI:** 10.1038/s41598-023-40451-8

**Published:** 2023-08-16

**Authors:** Michelle R. Bebber, Briggs Buchanan, Metin I. Eren, Robert S. Walker, Dexter Zirkle

**Affiliations:** 1 Department of Anthropology, Kent State University, Kent, OH 44242 USA; 2 Department of Anthropology, University of Tulsa, Tulsa, OK 74104 USA; 3 Department of Archaeology, Cleveland Museum of Natural History, Cleveland, OH 44106 USA; 4 Department of Anthropology, University of Missouri, Columbia, MO 65211 USA

**Keywords:** Archaeology, Cultural evolution, Anthropology

## Abstract

The atlatl is a handheld, rod-shaped device that employs leverage to launch a dart, and represents a major human technological innovation. One hypothesis for forager atlatl adoption over its presumed predecessor, the thrown javelin, is that a diverse array of people could achieve equal performance results, thereby facilitating inclusive participation of more people in hunting activities. We tested this hypothesis via a systematic assessment of 2160 weapon launch events by 108 people who used both technologies. Our results show that, unlike the javelin, the atlatl equalizes the velocity of female- and male-launched projectiles. This result indicates that a javelin to atlatl transition would have promoted a unification, rather than division, of labor. Moreover, our results suggest that female and male interments with atlatl weaponry should be interpreted similarly.

## Introduction

The invention of projectile hunting technology provided several advantages to past humans^1–5^. Relative to thrusting a handheld spear directly into a target, hunting with projectile weaponry increases the distance between human predator and animal prey. This gap would have reduced the chance of hunter injury, increased hunter concealment, and potentially increased weapon effectiveness^6,7^. Among the first projectile hunting weapons were thrown wooden javelins, some likely tipped with stone points^3,5,9–12^.

After javelins, past humans developed mechanically assisted projectile delivery systems, such as the atlatl and dart and the bow and arrow. The atlatl and dart is often assumed to be the first of these systems; a reasonable and parsimonious assumption given its production simplicity relative to the bow and arrow^1,13,14^. The atlatl is a straightforward yet sophisticated weapon delivery system consisting of a handheld, rod-shaped device which is used to propel a lightweight spear, referred to as a dart. Atlatls are made of wood, bone, antler, or a combination of these materials. They possess a handle on one end and a hook or indentation on the opposite end, which functions to engage the tail end of the dart.

Yet in some regions a transition from the javelin directly to the bow and arrow likely occurred^15–19^. Additionally, simultaneous use of different weapons has been documented ethnographically as well as suggested for some Paleolithic or forager societies^14,20–23^. The lack of consistent javelin, atlatl, dart, bow, and arrow preservation in the Pleistocene archaeological record impedes a clear understanding of technological trajectories in most geo-temporal contexts^8,14,24–26^. Regardless of archaeological obfuscation, it is reasonable to presume that transitions from thrown javelin to atlatl and dart occurred in at least some specific contexts during the human past. Achieving a better understanding of why that transition occurred is our focus here.

A transition from the thrown javelin to the atlatl-launched dart would have potentially involved tradeoffs in performance attributes and would have depended on ecology and prey characteristics, as well as economic, demographic, and cultural factors^15,27,28^. Launching a dart via an atlatl essentially employs a class 1 lever: force is applied by hand to the short arm of the lever, moving the dart at the long arm of the lever, with the wrist as a fulcrum in between^8^. Light-weight darts launched via an atlatl can thus, on average, achieve much faster speeds and farther distances than heavier, hand-thrown javelins^1,6,13,14,29^. Yet, recent controlled experiments suggest that despite a dart’s increase in velocity, a javelin’s heavier mass still results in a relatively larger kinetic energy^24^, which may in certain contexts have led to greater hunting success^7^. Additionally, while javelin use may have in some instances required closer proximity to prey, it may not have necessitated increased danger. For example, a person could throw javelins from trees^7^ or use them in environments with heavy vegetation which could increase concealment^25^.

One proposed hypothesis for the adoption of the atlatl and dart over the thrown javelin is that the former acted as an “equalizer”, thereby facilitating the participation of more people in a hunt, and reducing social disparity^27,30,31^. Researchers have proposed that the effective use of javelins would have required strength, a potentially larger body size, and high investment in training^23,32^. If the atlatl and dart render body size, strength, or training time less important to hunting success, then more, or different, people could partake in the hunt which would allow a greater number of tactical options, increase the chances of hitting the animal target, and reduce the onus of hunting success on a small number of individuals possessing a particular set of physical attributes.

If the atlatl was an equalizer, then there is a clear, testable prediction to support that hypothesis: in a large group of people using both a thrown javelin and the atlatl and dart, a segment of that group should see a disproportionate increase in performance in their atlatl and dart use relative to their javelin use such that any differences are eliminated. In other words, a new technology cannot be deemed an “equalizer” unless there is a documented inequality in the previous technology. Here, we test that prediction via a systematic assessment of 108 people who used both technologies. Our measured performance attribute was velocity. We predicted that if the atlatl acts as an equalizer, then a segment of our tested population who achieved relatively poor javelin velocities would exhibit a disproportionate increase in their atlatl-launched dart velocities and thus match the rest of the population.

## Results

**Figure 1:**
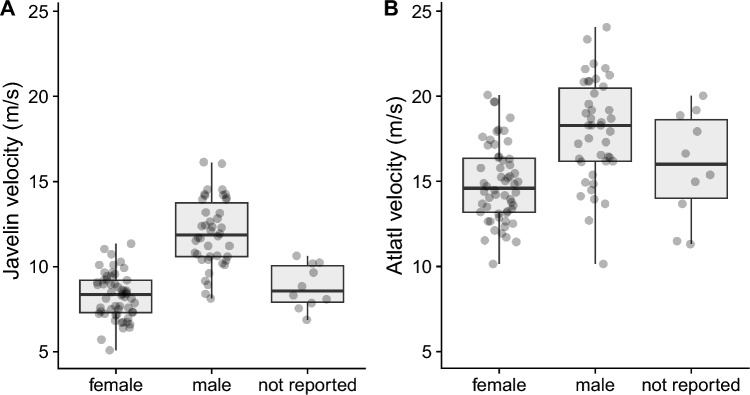
Box and whisker plot shows that, without conditioning on grip strength, female thrown javelin velocity is significantly less than thrown male javelin velocity, whereas mean female and male atlatl dart velocity overlap more substantially. The central horizontal line is the median, box is the interquartile range, and vertical whiskers are 95% of the distribution. (**A**) Thrown javelin velocity by sex, and (**B**) atlatl velocity by sex.

**Figure 2:**
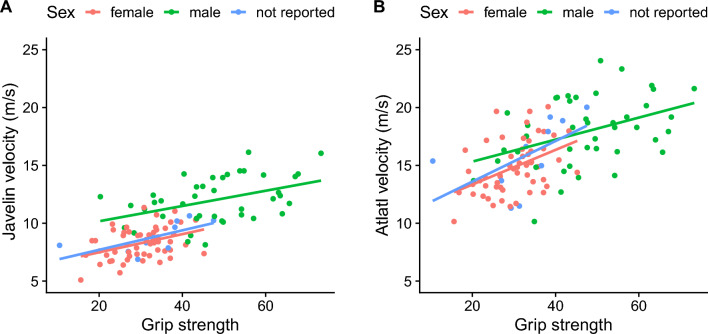
Scatterplots of individual grip strength and velocity. (**A**) thrown javelin velocity and (**B**) atlatl velocity. Points and best fit lines are colored by sex (red = female, green = male, blue = not reported).

We compared the mean velocity between hand thrown javelins and atlatl launched darts (see Supplementary Materials). There is a strong correlation (r^2^ = 0.74) between individual javelin and dart velocities (see Supplementary Fig. S1). Mean dart velocity (x̅ = 16.2 m/s) is 65% faster than mean javelin velocity (x̅ = 9.8 m/s). This result is consistent with previous work on these weapon delivery systems^23,24,33^. A box plot of javelin and dart velocity by sex shows that without conditioning on grip strength female javelin velocity is significantly less than male javelin velocity (the not reported category exhibits velocity similar to the female category), whereas there is more overlap between female and male dart velocity (Fig. 1). We also carried out exploratory data analysis examining the effect of age on javelin and dart velocity. The relationship is nonlinear with a peak velocity around 25 years of age (see Supplementary Fig. S2). The relationship between grip strength and javelin and dart velocity is linear (Fig. 2). In the bivariate plot of grip strength and dart velocity (Fig. 2b), the female (and the not reported category) data overlap more with the male data relative to the plot of javelin velocity (Fig. 2a).

**Figure 3:**
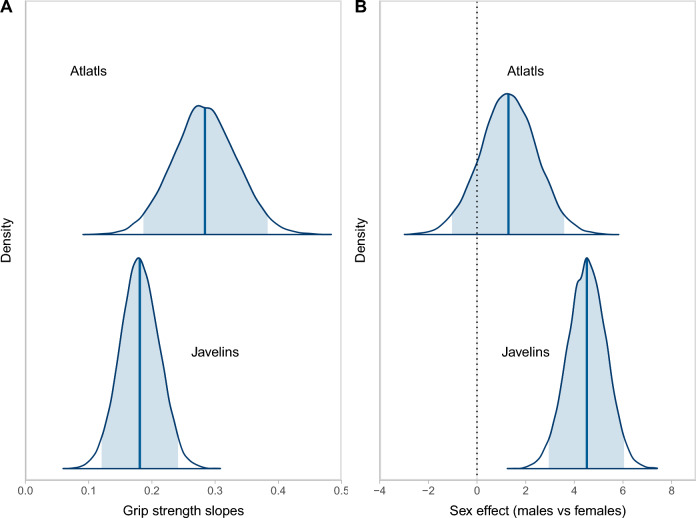
Conditional posterior distributions for slope estimates of thrown javelin and atlatl dart velocities. (**A**) The grip strength slopes while conditioning on sex and age. (**B**) The sex effect of males versus females while conditioning on grip strength and age. Note that the 95% credible interval (shaded) of the sex effect for thrown javelin velocity does not overlap zero and is significant, while the slope estimate for atlatl dart velocity does overlap zero and is not significant.

The full Bayesian structural equation model shows that javelin velocity and grip strength are significantly greater for males than females and not reported, whereas there are no significant differences among the male, female, and not reported categories in dart velocity when conditioned on grip strength and age (Table 1; Fig. 3). Grip strength remains a significant variable in dart velocity even though sex is nonsignificant, that is, while sex is unimportant in atlatl dart launching the variation in grip strength relates to dart velocity: the greater the grip strength the higher the velocity.

**Table 1:** Results of the full Bayesian structural equation model where the first term is the outcome and the second term is the predictor. (A) The smoothed age-related terms included in the model. (B) The population-level effects in the model including the intercepts and slopes for the terms. (C) The family specific parameters which are the variation terms. The female sex category is the baseline. *95% credible interval of the estimate does not include zero. “Javelin” refers to thrown spear; “Atlatl” refers to the atlatl dart.

	Estimate	Est. error	Lower-95% CI	Upper-95% CI
(A) Smooth terms				
Grip strength-age	4.66*	4.64	0.14	16.75
Atlatl velocity-age	2.71*	2.08	0.25	8.03
Javelin velocity-age	2.65*	1.59	0.54	6.54
(B) Population-level effects				
Grip strength-intercept	34.98*	1.22	32.56	37.37
Atlatl velocity-intercept	11.22*	0.77	9.70	12.73
Javelin velocity-intercept	6.02*	0.46	5.12	6.92
Grip strength-male	2.56*	0.98	0.59	4.47
Grip strength-sex NR	− 0.17	0.97	− 2.08	1.75
Atlatl velocity-male	0.62	0.54	− 0.43	1.68
Atlatl velocity-sex NR	0.29	0.64	− 0.93	1.55
Atlatl velocity-Grip strength	0.13*	0.02	0.08	0.17
Javelin velocity-male	2.08*	0.36	1.38	2.78
Javelin velocity-sex NR	0.15	0.44	− 0.71	1.01
Javelin velocity-Grip strength	0.08*	0.01	0.05	0.11
Grip strength-Age	0.02	1.00	− 1.93	1.97
Atlatl velocity-Age	− 0.11	1.00	− 2.04	1.84
Javelin velocity-Age	0.03	0.98	− 1.89	1.95
(C) Family specific parameters				
Sigma-grip strength	12.51*	0.91	10.49	14.07
Sigma-atlatl velocity	2.36*	0.17	2.06	2.72
Sigma-spear velocity	1.38*	0.10	1.19	1.59

## Discussion

A transition from one weapon system to another in the human past would have been influenced by many factors, and, in turn, would have subsequently impacted many aspects of Paleolithic and forager societies. Here, we experimentally examined a proposed, but until now untested, benefit of transitioning to atlatl launched darts from thrown javelins: namely that the atlatl and dart equalizes hunting performance among different segments of a population^27,30,31^. Our results are consistent with this “atlatl equalizer hypothesis”, showing that the atlatl not only increases the velocity of projectile weapons relative to thrown javelins, but that the atlatl equalizes the velocity of projectiles among different segments of a population. In other words, our results are consistent with the hypothesis that a transition from the thrown javelin to the atlatl and dart would have permitted a unification, rather than division, of labor by allowing more, and different, people to participate in hunting behaviors^30^.

Although female hunting has been documented in the ethnographic record^34–37^, no pattern in recent times should be automatically imposed onto the deep human past^30,38^. Yet, some archaeologists have entirely dismissed the possibility of past female hunting, which has led to unsupported and biased interpretations of archaeological data and context^39–41^. For example, more recently Hemmings^42^ assumed that at the Late Pleistocene Murray Springs Clovis site “a minimum of 22 ‘armed’ males” participated in the hunt. But when considering our velocity results together with those of Whittaker and Kamp^31^, who found modern female atlatl accuracy scores to be “close if not equal to men,” we propose that assumptions about gender, like that of Hemmings’^42^ are unsupported if, as he proposes, the atlatl were used (although we note that there is no direct evidence for Clovis atlatl use, see discussion in Refs.^8,14,25,43^). Instead, the practice of female hunting should be a prediction to be tested, or at least not prematurely disregarded, in archaeological contexts in which the atlatl is either documented as the dominant weapon system or inferred to be employed. Moreover, the negligible differences between female and male atlatl performance suggest differences in the archaeological interpretation of females and males interred with atlatl gear should be correspondingly negligible^30,37,44^. Indeed, Haas et al.^30^ extensively reviewed archaeological data from burial contexts containing weapon components associated with big game hunting and found that 40% of sexed individuals interred with hunting tools were female.

Speculatively, given that our experimental results suggest that females are the segment of the population that disproportionately benefit in the transition from the thrown javelin to the atlatl and dart, we feel that it is worth considering on a theoretical level that in at least some archaeological contexts females could have been the atlatl’s inventor. Hypothetically, females’ familiarity with the increased leverage that comes with longer digging sticks or children’s toys^45–49^, could have led to a transformation of these implements’ original functions into weaponry. The point of this speculation is not to say females definitively invented the atlatl, but instead to note that, like assumptions regarding gender and hunting, any assumptions regarding gender and atlatl innovation cannot currently be sustained.

Our results also speak to three immediate avenues of future research. The first is achieving a more precise comparative understanding of interacting biomechanics, technology, learning curves, and training times. Beyond vague notions of strength or body mass differences, or the employment of levers^8,23^, our experimental results suggest that researchers will need to better reconcile why exactly males are significantly better at javelin throwing (which is also evident in modern sport), and exactly how females lessen or eliminate this advantage with the atlatl. We note that javelin throwing involves a dynamic and robust transfer of energy that recruits major muscle groups of the lower body, trunk, and upper limbs^50,51^. Because 70% of javelin release speed is established in the last 0.1 s of the throwing motion^52^, future research will investigate the influence and extent of terminal trunk rotation between sexes. Secondly, the most common injury among modern javelin throwers is an insult to the medial ulnar collateral ligament^53,54^. Interestingly, female athletes more commonly present chronic ulnar collateral ligament injuries versus more acute insults in males^55^. Thus, future research will also examine if chronic ulnar collateral ligament injuries in female javelin throwers could have been a limiting factor for its use in women of the past and led to a sex-biased use of the atlatl. Lastly, both males and females exhibit an increase in velocity (m/s) with atlatl use versus javelin throwing. However, on average, the relative increase in velocity in males is less than in females (males =  + 6 m/s; females: + 7 m/s). We aim to expand on this and other issues to explore whether male atlatl velocity reaches a point of relative diminishing returns compared to females due to abundant force on the oscillatory properties of the projectile dart.

The second avenue of future research involves the construction and testing of predictions that speak to when past humans would have employed the atlatl relative to other weaponry options. For example, one might predict atlatl use over the bow and arrow under specific demographic circumstances, such as during dispersal events or other similar scenarios in which effective human population sizes are relatively small, decreasing, or isolated^56,57^. In this hypothetical scenario, relative to the bow and arrow, the atlatl would have allowed more people to participate in hunting, which would have increased hunting success and thus helped avoid further population loss, while its less complex production might have decreased the chance of technological loss. Of course, more variables should be considered in the construction of weaponry choice predictions^15^, and we emphasize that we have not tested gender performance differences related to the bow and arrow, but our point here is that experimental support for tool variant advantages or disadvantages can potentially strengthen our archaeological predictions and our understanding of the evolution of technology^58^.

The third avenue of future research involves the replication of our experiment. Replication should occur such that as many experimental variables and conditions as possible are similar to what we reported here, as well as systematic and strategic alteration of these variables and conditions. For example, male versus female use of heavier and lighter atlatl darts and javelins (relative to those we tested here) could be compared, as could the use of different atlatl types. Assessing males and females of varying atlatl and javelin skill, experience, and age relative to the tested participants in our study is also an important consideration for future research. Experimental conditions could also be altered, such as allowing a full running start for throwing the javelin or using the atlatl, or randomly have participants start with either the atlatl or the javelin. We also note that other atlatl and javelin performance criteria, such as accuracy, precision, or target penetration depth should eventually be compared between diverse individuals.

There are cultural, economic, and functional costs and benefits to different past weaponry systems. The experimental results reported here support a previously proposed, but until now untested, benefit to the atlatl: performance equalization among diverse individuals. This result is consistent with the broader hypothesis that, relative to the javelin, the atlatl—in some particular archaeological contexts—would have been an elegant, effective weaponry solution.

## Methods

**Figure 4:**
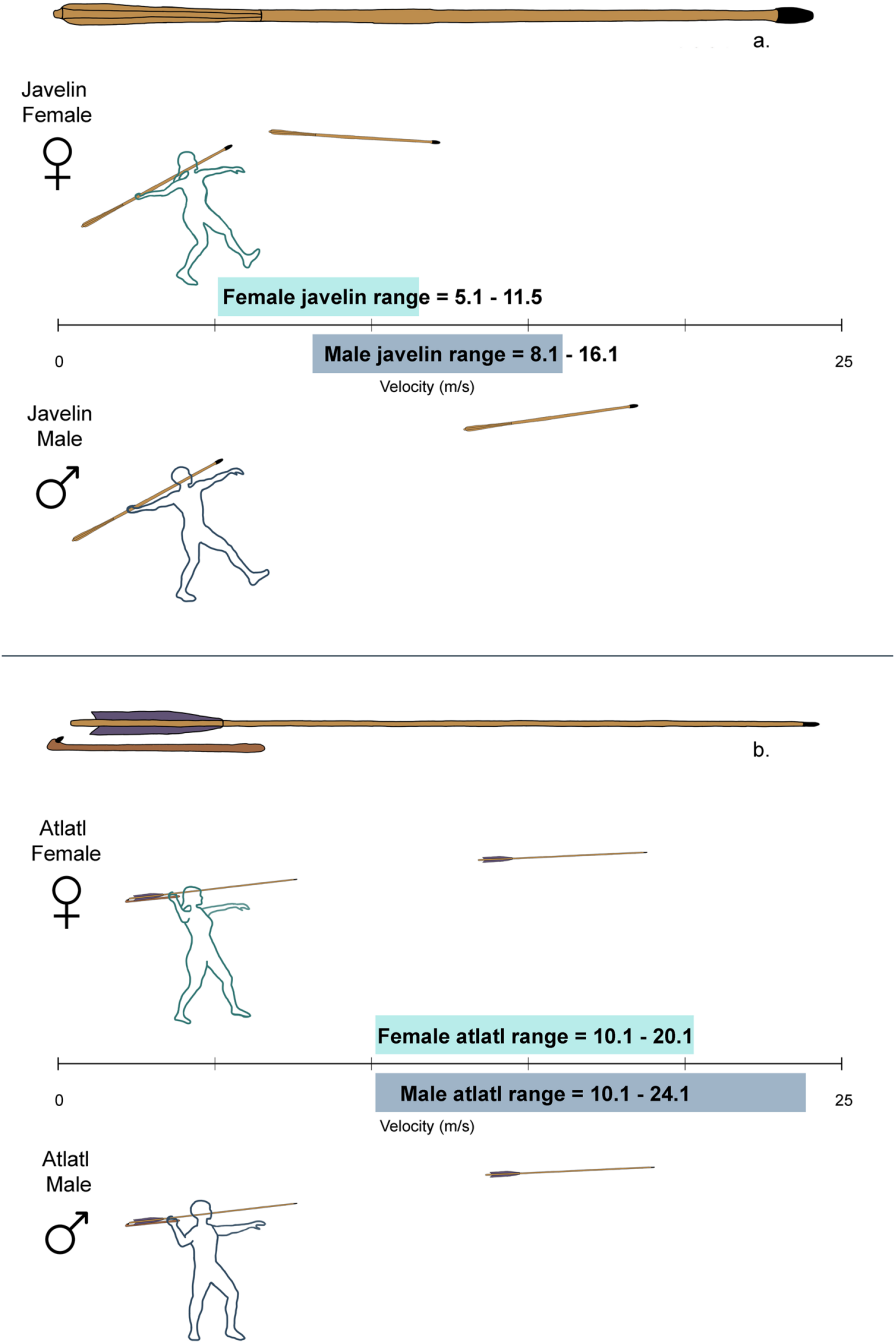
Our experiment employed modern javelins (**a**) and atlatls and darts (**b**). Each of the 108 participants launched a dart with an atlatl ten times and then threw a javelin ten times and for a total of 2160 weapon launch events. The average female atlatl velocity range of 10.1–20.1 m/s overlapped the average male atlatl velocity range of 10.1–24.1 m/s, while the female javelin velocity range of 5.1–11.5 m/s is significantly lower than the average male javelin velocity range of 8.1–16.1 m/s.

Our experiment employed modern javelins and atlatls and darts. The thrown javelins (Fig. 4a) were Turbojav brand training javelins with a length of 182 cm, a diameter of 3.5 cm, and a mass of 800 g. We selected this javelin because the overall shape, length, and mass are similar to those of the Schoningen spears^11^ and to spear replicas used in other experiments^23^.

The atlatls and darts (Fig. 4b) were supplied by Thunderbird Atlatl (thunderbirdatlatl.com) and are consistent with the Basketmaker style found in the southwestern USA. The atlatls were 66.5 cm long and 2.3 cm wide, with mass of 105 g. The darts used were 182 cm long, 1.2 cm in diameter, with a mass of 200 g. These experimental values are comparable to ethnographic ranges and those used in other experiments^33,59–62^.

Our study included 108 volunteer participants who used both technologies. All participants were from the Kent State University community and surrounding area. We collected biometric data that included age, height, weight, arm length, grip strength, pinch strength, and sex; all biometric data are available in the supplementary online materials. Age, height, weight, and sex were self-reported. We recorded arm lengths by measuring from the top of the shoulder joint to the wrist, bicep circumference by measuring around the bicep in a relaxed, unflexed position, grip strength via a Patterson Medical Jamar Plus Hand Dynamometer, and pinch strength via a Patterson Medical Jamar Digital Pinch Gauge. In total there were 42 males and 56 females, while 10 people did not disclose their biological sex. Participants ranged in age from 11 to 72 years old.

Our study was approved by ethical committee of Kent State University and deemed exempt from IRB review (IRB ID: R_2CVPc1DO1aZbpAr) on the basis that we collected no data covertly, the data were fully anonymized, and participants were not vulnerable. We orally briefed participants on the study procedures (but not on the study goals), and each participant signed an informed consent document. Participants were informed that they could withdraw at any stage of the study without penalty and their data would not be used. All research was performed in accordance with relevant regulations, and we confirm that informed consent was obtained from all participants and/or their legal guardian.

We provided each participant with a demonstration of how to throw the javelin and how to use an atlatl to launch a dart. Both procedures required that the participants remain more or less in place, with no running prior to launching a weapon^24^, but they were allowed up to three steps from their starting position. Participants were given up to 30 min to practice with each technology before velocity data collection commenced, but most practiced for less than 10 min. We told participants to launch their weapons at a foam target, which was placed 25 m away.

Each of the 108 participants first shot a dart with an atlatl ten times and then threw a javelin ten times for a total of 2160 weapon launch events. We then averaged each participant’s 10 javelin velocities and 10 dart velocities to produce a mean javelin velocity and mean dart velocity per participant. We measured velocity (recorded in mph and then converted to m/s) with a Bushnell “Velocity” radar gun positioned behind the thrower so that the radar gun was aimed at the line of travel of the projectile. The accuracy of this procedure has been demonstrated in prior experiments^33^.

To avoid any experience-bias, as well as the potential difficulty of quantifying experience and skill, we ensured each of our participants was naïve with respect to their use of both technologies. Nevertheless, our recorded data replicate the kinetic energy [KE] results of prior research^24^, where, in controlled experiments using two expert participants, and weaponry with different mass and morphometrics, it was demonstrated that thrown javelins yield higher KE values than darts shot with an atlatl. Prior results^24^ show thrown javelins exhibit average KEs of 74.2 J at 4 m and 71.7 J at 10 m, while darts exhibit average KEs of 55.2 J at 4 m and 43.9 J at 10 m. When these latter expert results are compared to the mean javelin and dart KEs produced by our 108 naïve participants, our results are predictably less in terms of absolute value, but comparatively analogous at 40.5 J for mean javelin KE (n = 108) and 27.0 J for mean dart KE (n = 108). It is also worth noting that the javelin and dart KE ranges demonstrated by a prior study^24^, overlap with our javelin and dart KE ranges (Prior study^24^ javelin at 4 m: 63.3–87.6 J; Prior study ^24^ javelin at 10 m: 60.4–89.3 J; present study javelin: 10.4–104.2 J; Prior study^24^ dart at 4 m: 45.0–61.5 J; Prior study^24^ dart at 10 m: 40.3–50.4 J; present study dart: 10.3–57.8 J).

**Figure 5:**
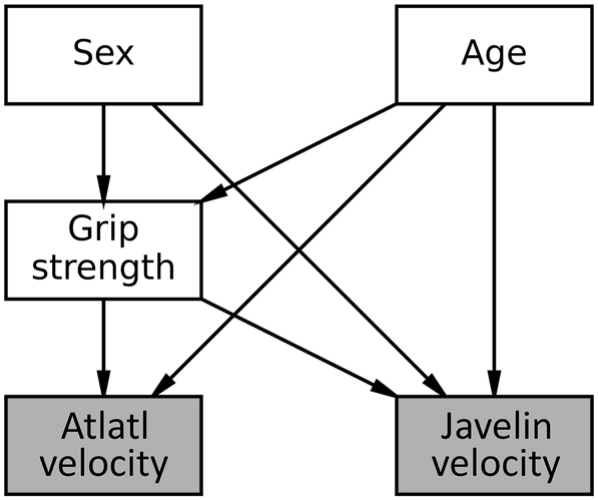
Directed acyclic graph (DAG) of the empirical measurements from the thrown javelin (spear) and atlatl dart experiment. Thrown javelin (spear) and atlatl dart velocity are outcomes of gender and age and mediated by grip strength. Not shown is the nonsignificant causal arrow between gender and atlatl dart velocity.

To link our experimental design to our empirical measures to make causal inference we use a directed acyclic graph (DAG). A DAG represents the conceptual causal connections between theory and our empirical data (or the empirical estimands). In the DAG (Fig. 5), the nodes or the rectangular boxes are variables, and the arrows represent causal associations. Note that in a DAG the causal flow is only in one direction and causal feedback or loops are not allowed. Operating with the assumption that our DAG is a causal DAG, we can assess which variables are included in the model and which need to be conditioned on to evaluate the effect of gender on javelin and atlatl dart velocity. In this case, both gender and age are common causes of grip strength and javelin velocity, and age has a causal association with grip strength and atlatl dart velocity (Supplementary Fig. S2). Because we are interested in the causal association between gender and javelin velocity and gender and dart velocity, we condition on grip strength in both cases as it is the mediator in the causal chain between gender and javelin and dart velocity. Similarly, we also condition on age because it has an association with grip strength and javelin and dart velocity.

### Supplementary Information

Supplementary Information 1.

Supplementary Information 2.

Supplementary Information 3.

## Data Availability

All data and code are available in the main text or the supplementary materials.
