# Supplementary material for: Atlatl use equalizes female and male projectile weapon velocity
Source: Sci Rep. 2023 Aug 16;13:13349. doi: 10.1038/s41598-023-40451-8 (PMC10432391; doi:10.1038/s41598-023-40451-8)
Supplement: Supplementary file 3 — Supplementary Information 3. [file 41598_2023_40451_MOESM3_ESM.docx]

Supplementary Materials for

Title: Atlatl use equalizes female and male projectile weapon velocity

**Authors:** *Michelle R. Bebber^1^, Briggs Buchanan^2^, Metin I. Eren^1,3^, Robert S. Walker^4^, Dexter Zirkle^1^

*Corresponding author. Email [mbebber@kent.edu](mailto:mbebber@kent.edu)

**The supplemental PDF file includes:**

Supplementary Text

Figs. S1 to S2

Table S1

**Other Supplementary Materials for this manuscript include the following:**

Data S1

Atlatl.R

Supplementary Text

In the Results section we report the results of a ‘Bayesian estimation supersedes the t-test’ (BEST) model comparing javelin and atlatl velocities (Figure S1). BEST models are Bayesian equivalents to t-tests and utilize the student’s t family of distributions (*63*). Our distributional BEST models allow unequal variance between groups and are run in R 4.1.2 (R Core Team) with the brms package. Following Kruschke (*63*), we used a weak exponential prior with a rate of 1/29 for nu, the parameter that changes the normality of the distribution (i.e., the degrees of freedom parameter in the t distribution) and a logarithmic prior for the variance, or sigma, in each group. Sampling was carried out using the No-U-Turn Sampler (NUTS) (*64*). Final models were run with four chains for 10,000 iterations with a ‘warm-up’ of 5,000 iterations and 20,000 total post-warmup draws. For all parameters, r-hat values (a model diagnostic with expected value equal to 1) were exactly 1.00 and hence confirm model convergence. Chains were also inspected visually for sufficient mixing to ensure that model results were appropriate. We used posterior distributions to make inferences about the strength of the group effects in the model. All data and R scripts are available in the supplementary online materials.

The BEST model results indicate that atlatl velocity is significantly higher than javelin velocity (Table S1). There is also more variation in the atlatl velocities.

Supplementary References:

63. J. Kruschke, Bayesian Estimation supersedes the t test. *J. Exp. Psychol.* **142**, 573–603 (2013).

64. M. D. Hoffman, A. Gelman, The No-U-Turn sampler: Adaptively setting path lengths in Hamiltonian Monte Carlo. *J. Mach. Learn. Res.*, 15(1):1593-1623 (2014).

**Fig. S1.** **Bivariate scatterplot of javelin velocity (m/s =meters per second) and atlatl velocity**. Points and best fit lines are colored by sex.

**Fig. S2.** **Scatterplots of individual age** **and velocity.** (**A**) javelin velocity and (**B**) atlatl velocity. Points and best fit lines are colored by sex. There is a nonlinear association between age and javelin and atlatl velocity. A peak in velocity in both delivery technologies is seen around age 20 and then decreases with greater age.

**Table S1**. **Results of the BEST Bayesian model.** (**A**) the population-level effects in the model including the intercepts and slopes for the terms. (**B**) the family specific parameters which are the variation terms. * 95% credible interval of the estimate does not include zero.

(A) Population-level effects:

|  | **Estimate** | **Est. error** | **Lower-95% CI** | **Upper-95% CI** |
| --- | --- | --- | --- | --- |
| Atlatl | 16.19 | 0.29 | 15.62 | 16.76* |
| Javelin | 9.77 | 0.23 | 9.32 | 10.24* |
| Sigma-atlatl | 1.08 | 0.07 | 0.94 | 1.23* |
| Sigma-javelin | 0.85 | 0.07 | 0.71 | 0.99* |

(B) Family specific parameters:

|  | **Estimate** | **Est. error** | **Lower-95% CI** | **Upper-95% CI** |
| --- | --- | --- | --- | --- |
| nu | 49.76 | 32.48 | 11.51 | 134.20* |

Data S1. (separate file)

Data file including velocity (mph and mps), kinetic energy (KE), and biometric measurements.

Atlatl.R (separate file)

Atlatl.R file contains all R scripts used in this study.
